# Association of resting-state theta–gamma coupling with selective visual attention in children with tic disorders

**DOI:** 10.3389/fnhum.2022.1017703

**Published:** 2022-09-29

**Authors:** Ji Seon Ahn, Kyungun Jhung, Jooyoung Oh, Jaeseok Heo, Jae-Jin Kim, Jin Young Park

**Affiliations:** ^1^Institute of Behavioral Science in Medicine, Yonsei University College of Medicine, Seoul, South Korea; ^2^Department of Psychiatry, Yonsei University College of Medicine, Yongin Severance Hospital, Yonsei University Health System, Yongin, South Korea; ^3^Center for Digital Health, Yongin Severance Hospital, Yonsei University Health System, Yongin, South Korea; ^4^Department of Psychiatry, International St. Mary's Hospital, Catholic Kwandong University, Incheon, South Korea; ^5^Department of Psychiatry, Yonsei University College of Medicine, Gangnam Severance Hospital, Yonsei University Health System, Seoul, South Korea; ^6^Department of Laboratory Medicine, Yongin Severance Hospital, Yonsei University Health System, Yongin, South Korea

**Keywords:** tic disorder (TD), electroencephalography, theta-gamma coupling, neuronal oscillations, resting state, selective attention

## Abstract

A tic disorder (TD) is a neurodevelopmental disorder characterized by tics, which are repetitive movements and/or vocalizations that occur due to aberrant sensory gating. Its pathophysiology involves dysfunction in multiple parts of the cortico-striato-thalamo-cortical circuits. Spontaneous brain activity during the resting state can be used to evaluate the baseline brain state, and it is associated with various aspects of behavior and cognitive processes. Theta–gamma coupling (TGC) is an emerging technique for examining how neural networks process information through interactions. However, the resting-state TGC of patients with TD and its correlation with cognitive function have not yet been studied. We investigated the resting-state TGC of 13 patients with TD and compared it with that of 13 age-matched healthy children. The participants underwent resting-state electroencephalography with their eyes closed. At the global level, patients with TD showed a significantly lower resting-state TGC than healthy children. Resting-state TGC with the eyes closed was significantly negatively correlated with the attention quotient calculated for omission errors in a selective visual attention test. These findings indicate that the resting-state brain network, which is important for the attentional processing of visual information, is dysfunctional in patients with TD. Additionally, these findings support the view that TGC reflects information processing and signal interactions at the global level. Patients with TD may have difficulty gating irrelevant sensory information in the resting state while their eyes are closed.

## Introduction

In recent years, there has been an increasing tendency to evaluate dysfunction in interactions within and between large brain networks rather than local dysfunction in single brain regions (Ramkiran et al., 2019; Openneer et al., 2020). Furthermore, it is becoming increasingly important to understand complex neurodevelopmental disorders as network problems (Stern et al., 2000; De La Fuente et al., 2013; Ramkiran et al., 2019). A tic disorder (TD) is a neurodevelopmental disorder characterized by repetitive, seemingly uncontrollable, out of context, and exaggerated motor and/or phonic tics (Leckman and King, 1999; American Psychiatric Association., 2013). These atypical features have been hypothesized to be sometimes caused by deficits in inhibition and top-down cognitive control (Jurgiel et al., 2021). The neural process of top-down modulation serves as a fundamental physiological mechanism for selective attention (Gazzaley et al., 2005; Gazzaley and Nobre, 2012). It relies on long-range inputs from and interactions with a network of control regions, including the prefrontal cortex and parietal cortex (Schmidt et al., 2002; Gazzaley and Nobre, 2012). Interestingly, these areas are involved in gating information and regulate motor and cognitive activity. In fact, the association of dysfunction of a large-scale neuronal network with TD has been repeatedly reported, and it can reflect immature functional brain organization in patients with TD (Church et al., 2009; Neuner et al., 2014; Fan et al., 2018; Wen et al., 2018). Church et al. (2009) observed an abnormal pattern of functional connections in the frontoparietal network of children with TD relative to age-matched healthy children (Church et al., 2009). Worbe et al. (2015) observed altered structural connectivity of cortico–basal ganglia networks (Worbe et al., 2015; Wen et al., 2018) observed disrupted default mode networks of children and adolescents with TD relative to healthy children (Wen et al., 2018). The default mode network has been defined as a network of interacting brain regions that is active during resting state activity and inactive during information processing or goal-directed tasks. It appears to reflect the spontaneous cognitive processes (Andrews-Hanna et al., 2010) involved in attention, memory, and thinking (Kounios et al., 2008). Cross-frequency coupling is emerging as a fundamental feature of brain activity and considered to be the mechanism underlying cortical information gating and processing, which enables conscious awareness and various cognitive functions (Lisman and Buzsáki, 2008). It can measure functional connectivity beyond single-frequency assessments of oscillatory activity and provide insight into the ways that local neural networks process information through interaction or coupling of activities across frequencies (Morillas-Romero et al., 2015; Voytek et al., 2015). Our previous study on the spatial patterns of cross-frequency phase–amplitude coupling between the theta and gamma bands showed an anticorrelation between the regions active in the resting state and those active while performing a visual working memory (WM) task. It indicated that theta–gamma coupling (TGC) reflects information processing and signal interactions between distant brain regions by reflecting functional interaction patterns between active brain regions (Ahn, 2022; Ahn et al., 2022). TGC, a type of cross-frequency coupling, is a neurophysiologic process during which the power of gamma oscillations is systemically modulated over the course of the theta cycle; it is the mechanism underlying perception, attention, learning, and WM (Axmacher et al., 2010; Park et al., 2013; Goodman et al., 2018). Selective attention refers to the processes that enable a person to orient the focus of conscious awareness toward relevant stimuli while inhibiting irrelevant or competing stimuli to limit what is encoded and stored in the WM (Gazzaley and Nobre, 2012). Resting-state brain activity has been associated with various aspects of behavior and cognitive processes and can be used to evaluate baseline brain states (Barry et al., 2010; Berman et al., 2015). Nevertheless, few studies have examined cross-frequency interaction in the resting state and its correlation with selective attention or WM. This study aimed to investigate resting-state functional interaction patterns in patients with TD using scalp electroencephalography (EEG) and compare the findings with those of age-matched healthy children, to improve the understanding of neural circuit coordination in patients with TD, and to identify neural correlates with selective visual attention (SVA) and selective auditory attention (SAA). To achieve this, we compared the resting-state EEG findings of patients with TD with those of age-matched healthy children and performed a comparative analysis of TGC during inter-tic intervals with the participant remaining as still as possible. Finally, relationships of the modulation index (MI) of resting-state TGC with the SVA and SAA quotients were analyzed. We hypothesized that the functional interaction patterns of children with TD would differ from those of healthy children in the resting state. Moreover, we expected that any observed aberrant resting-state functional interactions in patients with TD would indicate impairments in the ability to suppress competing attentional demands and respond appropriately, thereby reflecting the cognitive or self-regulatory function of patients with TD.

## Materials and methods

### Participants

Patients with TD diagnosed by experienced psychiatrists based on the criteria of the Structured Clinical Interview for the Diagnostic and Statistical Manual of Mental Disorders, fifth edition, were recruited from the outpatient unit of the Psychiatry Department of Catholic Kwandong University, International St. Mary's Hospital. Healthy children with no personal or family history of TD and no personal history of mental disorders, including attention-deficit/hyperactivity disorder and obsessive-compulsive disorder, were recruited through posters at the hospital. The inclusion criteria were age 6–18 years and ability to complete all required study procedures, as determined by a psychiatrist. The exclusion criteria were head trauma or brain lesions, diagnosis of a neurological disorder, estimated full-scale intelligence quotient < 70, and inability or unwillingness to provide consent for study participation. In addition, healthy children who used psychoactive medication were excluded. Each participant or their parent provided written informed consent to participate in the study after being explained the purpose and procedures involved. This study was reviewed and approved by the Institutional Review Board of Catholic Kwandong University International St. Mary's Hospital (No. IS21OISI0016).

### Clinical and cognitive assessments

All participants underwent psychiatric interviews and screening tests. Psychiatric conditions were diagnosed using the Kiddie Schedule for Affective Disorders and Schizophrenia-Present and Lifetime version, administered by either a psychiatrist or trained and supervised graduate-level psychologists. The Kiddie Schedule for Affective Disorders and Schizophrenia-Present and Lifetime version is a semi-structured interview based on the Diagnostic and Statistical Manual of Mental Disorders, fourth edition, criteria. It has been used to assess the severity of symptoms of 32 child and adolescent psychiatric disorders included in the Diagnostic and Statistical Manual of Mental Disorders, fourth edition (Kaufman et al., 1997). Subtests of the computerized Comprehensive Attention Test (CAT) were administered to evaluate SVA and SAA. Two outcome measures, omission errors (OEs) and commission errors (CEs), were quantified for each test. OE was defined as failure to respond to a target; it was calculated based on the number of missing target responses. OEs are related to attention processes, and lower scores indicate inattention. CE was defined as an inappropriate response to a non-target; it is thought to be influenced by impulsivity and hyperactivity, and lower scores indicate poor impulse control. The attention quotient (AQ) was determined for each parameter (OE and CE) based on age- and sex-matched normative data, assuming that the average AQ of the reference population was 100 (standard deviation, 15) (Kim et al., 2011, 2012).

### Instructions to participants

The patients with TD discontinued all medication for at least 48 h before participating in the experiment. Patients with TD were instructed to maintain a natural and comfortable state at rest without suppressing their tics. Each participant underwent EEG while seated comfortably in a chair in an electrically shielded and sound-attenuated room. Participants were encouraged to relax their jaw muscles and minimize ocular and body movements (e.g., they were asked to try to not blink or move their eyes). Before the beginning of the procedure, the participants were instructed to close their eyes, sit still on the chair, relax, and remain awake during the 5-min period.

### EEG data

#### EEG data acquisition

The EEG data were continuously recorded using Net station version 5.4 software (Electrical Geodesics, Eugene, OR, USA) and a 64-channel HydroCel Geodesic Sensor Net (Electrical Geodesics Inc., Eugene, OR, USA) based on the modified international 10–20 system of electrode placement, which is also called the 10–10 system (Applied Neuroscience, St. Peterburg, FL, USA). All EEG channels were referenced to the vertex electrode (Cz). Sponge-based carbon fiber electrodes (Ag/AgCl-coated and carbon-filled plastic electrodes with a sponge) were placed on the scalp in a high-density array. Before the sensor net was applied, the sponges were soaked in a solution of 5 mL/L of baby shampoo and 6 mL KCl/L of distilled water to facilitate electrical contact between the scalp and the electrodes. All electrode impedances were maintained at < 50 kΩ according to the guidelines of Electrical Geodesics Inc. The EEG data were digitized and amplified at a sampling rate of 1 kHz using the Geodesic EEG system 400 (Electrical Geodesics, Inc.) and filtered online using a bandpass filter set at 0.1–100 Hz and a notch filter set at 60 Hz (Ahn, 2022; Ahn et al., 2022).

#### EEG data preprocessing

The EEG data were preprocessed and analyzed offline using custom-written scripts (MATLAB 2016b; The MathWorks, Natick, MA, USA) and the EEGLAB toolbox (Delorme and Makeig, 2004). The Harvard Automated Processing Pipeline for EEG (HAPPE) (Gabard-Durnam et al., 2018) was used to automate preprocessing and artifact correction using a wavelet-enhanced independent component analysis and a multiple artifact rejection algorithm (Winkler et al., 2014). EEG recordings from children with neurodevelopment disorders have high degree of artifact contamination. HAPPE was designed for data with high levels of artifact or very short recording length (Gabard-Durnam et al., 2018). The continuous segment of the tic behavior-free periods during recordings were selected for analysis. Patients with TD did not always tic, and there were tic-behavior free intervals without a distinct tic when resting-state. The continuous EEG data were re-referenced to an average reference and filtered with a 1- to 240-Hz bandpass filter and 60-, 120-, 180-, and 240-Hz notch filters. Bad channels with error probability values that were < 3 standard deviations from the mean were removed. The bad channels were evaluated twice per data file. The data of these bad channels, which were removed, were interpolated from nearby channels during a later processing step. The signals of all channels removed during the bad channel rejection processing step were subjected to spherical interpolation with Legendre polynomials up to the seventh order. An independent component analysis was performed using a multiple artifact rejection algorithm in EEGLAB to eliminate components with artifact probabilities more than 0.8. Independent component analysis has been demonstrated to reliably isolate ocular, electromyographic, and electrocardiographic artifacts. The processed clean data were saved in the EEGLAB file format (.set files). Nineteen electrode sites among 57 channels were analyzed (Fp1, Fp2, F7, F3, Fz, F4, F8, T7, C3, Cz, C4, T8, P7, P3, Pz, P4, P8, O1, and O2) based on the standard international 10–20 system (Ahn, 2022; Ahn et al., 2022).

#### Power spectra analysis

The spectral power of the EEG data was calculated for each participant using fast Fourier transformation and the signal processing toolbox in MATLAB. Time windows of 4,000 ms with an 8-ms overlap were used for the spectral analysis. The absolute powers of the theta and gamma bands were averaged over all the time windows and frequency bands for further analyses (Ahn, 2022; Ahn et al., 2022). The following frequency bands were defined for spectral analyses: theta, 4–8 Hz, and gamma, 30–50 Hz.

#### TGC analysis

The intensity of cross-frequency coupling between the phase of the 4–8 Hz theta oscillations and the amplitude of the 30–50 Hz gamma oscillations was analyzed to determine the MI (Tort et al., 2010). The MI measures the divergence of the phase-amplitude distribution from the uniform distribution (MI = 0). The further the MI value is from 0, the lower the entropy and the greater the coupling. To obtain the MI value, phase-amplitude coupling analysis was performed on 20–200 s of clean data (5–50 s epoch). To calculate the MI of TGC, the standard Hilbert transform was applied to obtain the time series of the phase of the theta band and the amplitude envelop of the gamma band. Next, the composite time series was constructed, which provides information on the gamma oscillation at each phase of the theta rhythm. The theta phase was sorted into 72 5-degree bins spanning from −180° to 180°, degree interval and the corresponding mean amplitudes of the gamma oscillations were calculated for each phase bin. Finally, they were normalized by dividing the mean amplitude of each phase by the sum of all bins. The existence of PAC is characterized by a deviation of the amplitude distribution P from the uniform distribution in a phase-amplitude plot (Tort et al., 2010; Ahn et al., 2022). The MI was calculated as follows (Tort et al., 2009, 2010):


$$\begin{array}{l} MI=\frac{\log\left(N\right)-H\left(P\right)}{\log\left(N\right)} \end{array}$$

Here, N is the number of phase bins, log (N) represents the entropy of a uniform distribution, and H(P) is the entropy of P (Tort et al., 2010; Ahn et al., 2022).

### Statistical analysis

The demographic and clinical variables of the patients with TD and healthy children were compared. Continuous variables were compared using the independent Student's *t*-test or Mann–Whitney *U*-test. Attention quotients on individual CAT subtests in patients with TD and healthy children were compared by two sample independent Student's *t*-tests, with Bonferroni's correction for multiple comparisons. Dichotomous variables were compared using the chi-square test or Fisher's exact test as appropriate, depending on the distribution. Independent *t*-tests with the false discovery rate (FDR) correction were conducted to examine differences in the absolute theta power between patients with TD and healthy children. Similarly, independent *t*-tests with the FDR correction were conducted to examine differences in the resting MI of TGC between patients with TD and healthy children. Pearson correlation coefficients were determined after applying FDR correction to the significant findings obtained from the aforementioned analyses to examine the correlation between EEG findings and neuropsychological outcomes. To address the multiple comparisons problem, the FDR, assessed using the Benjamini–Hochberg method (Benjamini and Hochberg, 1995), was applied to all 19 electrodes used to obtain the individual EEG data of the participants. Multiple hypothesis testing is concerned with controlling the rate of false positives when testing several hypotheses simultaneously (Ebrahimi, 2008). The appropriate measure to take into account the multiple testing problem is the q value, which is the FDR adjusted *p*-values obtained from methods such as the Benjamini–Hochberg (Benjamini and Hochberg, 1995) and Storey's (Storey, 2002). The methods are based on the assumption that the distribution of *p*-values corresponding to null hypotheses follows a uniform distribution between zero and one (Hackstadt and Hess, 2009). A measure of significance in terms of the FDR can be calculated, producing individual q values. A test's q value is the minimum FDR at which the test would be declared significant. The q value maintains power by allowing the investigator to achieve an acceptable level of true or false positives within the calls of significance (Storey, 2003; Karp et al., 2007). Relationships between EEG parameters and clinical variables were evaluated using linear regression models with age and sex as covariates. Statistical significance was set at *p* < 0.05 (two-tailed). The MATLAB 2016b (MathWorks, Natick, MA, USA) statistical toolbox and Statistical Package for the Social Sciences version 25.0 (IBM Corp., Armonk, NY, USA) software were used for all statistical analyses.

## Results

Thirteen right-handed patients with TD (12 boys; 92.3%) whose ages ranged from 7 to 16 years (mean age 10.2 ± 3.9 years) and 13 right-handed healthy children (7 males; 53.8%) whose ages ranged from 6 to 18 years (mean age 10.8 ± 3.2 years) were included in this study. Their clinical characteristics and CAT subtest results are described in Table 1. There were no significant between-group differences in age and intelligence quotient. The proportion of boys in the TD group was higher than that in the control group, but this difference was not statistically significant (Fisher's exact test, *p* = 0.073). The CAT subtest results showed that the AQ of OEs for SVA of patients with TD higher than that of healthy children (104.5 ± 6.3 in patients with TD, 93.3 ± 13.2 in healthy children; *t* = −2.04; *p* = 0.013), whereas the AQ of CEs for SVA of healthy children higher than that of patients with TD (98.5 ± 14.8 in patients with TD, 99.9 ± 24.4 in healthy children; *t* = 0.19; *p* = 0.855). On the SAA test, the mean AQ of OEs (109.7 ± 10.4 in patients with TD, 100.9 ± 10.8 in healthy children; *t* = −0.72; *p* = 0.046) and CEs (100.9 ± 20.6 in patients with TD, 98.2 ± 19.2 in healthy children; *t* = −0.51; *p* = 0.740) in patients with TD both higher than that of healthy children. The Bonferroni's corrected significance level for 4 comparisons was *p* < 0.0125. None of the comparisons were statistically significant after Bonferroni's correction (adjusted alpha *p* < 0.0125 for *t*-tests).

**Table 1:** Demographic and clinical characteristics of the patients with TD and HC.

**Variables**	**TD**	**HC**	***T***	***p***
	**Mean (SD) or** ***N*** **(%)**			
Age, years	10.2 (3.9)	10.8 (3.2)	0.39	0.702
Male, *n* (%)	12 (92.3)	7 (53.8)		0.073
IQ	102.1 (14.4)	104.5 (19.4)	0.36	0.726
Medication, *n* (%)	7 (53.8)			
**Comorbidity**
None, *n* (%)	6 (46.2)			
ADHD, *n* (%)	5 (38.5)			
Depression, *n* (%)	2 (15.4)			
**CAT subtests**
SVAQ OE	104.5 (6.3)	93.3 (13.2)	−2.04	0.013
SVAQ CE	98.5 (14.8)	99.9 (24.4)	0.19	0.855
SAAQ OE	109.7 (10.4)	100.9 (10.8)	−0.72	0.046
SAAQ CE	100.9 (20.6)	98.2 (19.2)	−0.51	0.740

TD, tic disorder; HC, healthy children; SD, standard deviation; ADHD, attention-deficit/hyperactivity disorder; FSIQ, full-scale intelligence quotient; CAT, Comprehensive Attention Test; SAAQ, selective auditory attention quotient; OE, omission error; SVAQ, selective visual attention quotient; CE, commission error.

**Figure 1:**
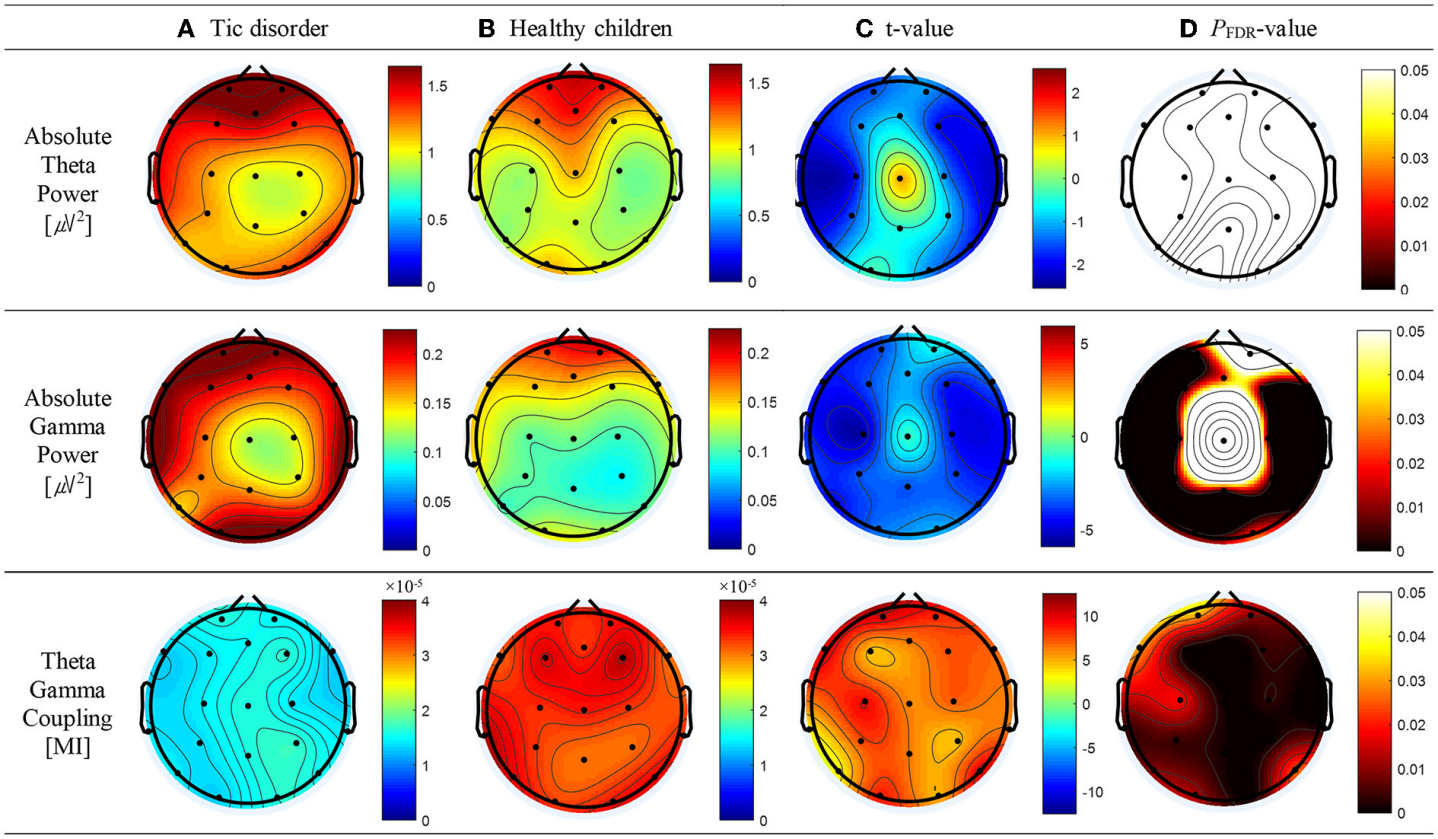
Topographic maps of absolute theta power, absolute gamma power, and theta–gamma coupling (TGC). **(A)** Absolute theta power, absolute gamma power, and TGC in patients with tic disorder. **(B)** Absolute theta power, absolute gamma power, and TGC in healthy children. **(C)** Topographical distribution of the *t*-value. **(D)** Topographical distribution of the corresponding false discovery rate–corrected *p*-value.

Compared with healthy children, patients with TD had a greater absolute theta power at all electrodes except Cz; however, significant differences below the FDR threshold were not observed. The topographic maps of the resting-state absolute theta power of patients with TD and healthy children are shown in Figures 1A,B, top panel, respectively. Patients with TD had significantly greater absolute gamma power than healthy children at the Fz (*t* = 2.46; *p* = 0.02), FP1 (*t* = 3.71; *p* = 0.001), F3 (*t* = 3.68; *p* = 0.001), F7 (*t* = 4.34; *p* = 0.01), C3 (*t* = 5.46; *P* < 0.001), T3 (*t* = 3.66; *p* = 0.001), P3 (*t* = 3.64; *p* = 0.002), T5 (*t* = 4.48; *p* = 0.001), Pz (*t* = 3.54; *p* = 0.002), O1 (*t* = 3.24; *p* = 0.004), O2 (*t* = 2.55; *p* = 0.019), P4 (*t* = 3.88; *p* = 0.001), T6 (*t* = 3.70; *p* = 0.01), C4 (*t* = 4.44; *p* = 0.001), T4 (*t* = 4.49; *p* = 0.001), F8 (*t* = 3.37; *p* = 0.003), and F4 (*t* = 4.31; *p* = 0.001) electrodes; the *p*-values for these differences were below the FDR threshold. The topographic maps of the resting-state absolute gamma power of patients with TD and healthy children are shown in Figures 1A,B, middle panel, respectively. Patients with TD had a significantly lower MI of TGC than healthy children at the FP2 (*t* = 8.26; *p* = 0.01), Fz (*t* = 6.27; *p* < 0.001), FP1 (*t* = 10.08; *p* = 0.03), F3 (*t* = 4.55; *p* < 0.001), F7 (*t* = 10.14; *p* = 0.03), C3 (*t* = 9.10; *p* = 0.018), T3 (*t* = 3.36; *p* = 0.004), P3 (*t* = 7.33; *p* = 0.004), T5 (*t* = 2.91; *p* = 0.009), Pz (*t* = 6.25; *p* < 0.001), O1 (*t* = 8.49; *p* = 0.012), O2 (*t* = 4.95; *p* < 0.001), P4 (*t* = 4.56; *p* < 0.001), T6 (*t* = 9.57; *p* = 0.024), C4 (*t* = 6.96; *p* = 0.003), T4 (*t* = 6.49; *p* = 0.002), F8 (*t* = 6.78; *p* = 0.002), F4 (*t* = 7.18; *p* = 0.004), and Cz (*t* = 5.91; *p* < 0.001) electrodes; the *p*-values for these differences were below the FDR threshold. The topographic maps of the resting-state MI of TGC of patients with TD and healthy children are shown in Figures 1A,B, bottom panel, respectively. The results are summarized in Supplementary Table 1.

**Figure 2:**
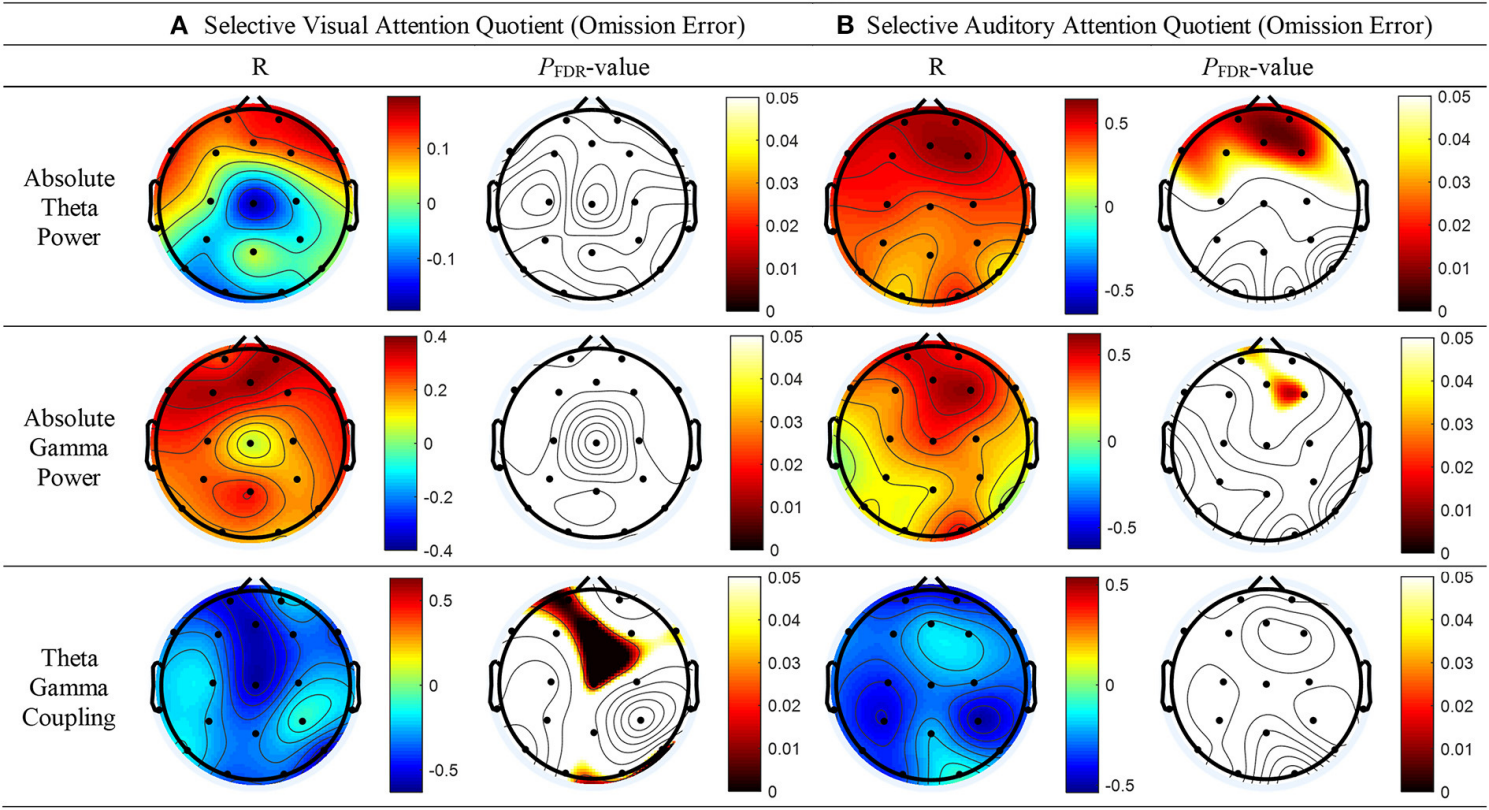
Topographical representation of the results of the Pearson's correlation analysis conducted to determine whether absolute theta power, absolute gamma power, and theta–gamma coupling are associated with the attention quotients (AQs) of omission errors (OEs) for selective attention in patients with tic disorder and healthy children. **(A)** AQs of OEs for selective visual attention (left panel: Pearson's correlation coefficients, right panel: false discovery rate–corrected *p*-values). **(B)** AQs of OEs for selective auditory attention (left panel: Pearson's correlation coefficients, right panel: false discovery rate–corrected *p*-values).

As shown in Figure 2B, top panel, a significant correlation was found between the absolute theta power and the AQ of OEs for SAA at the FP2 (*r* = 0.57; *p* = 0.013), Fz (*r* = 0.60; *p* = 0.012), FP1 (*r* = 0.56; *p* = 0.013), F3 (*r* = 0.49; *p* = 0.034), F7 (*r* = 0.53; *p* = 0.021), and F4 (*r* = 0.61; *p* = 0.012) electrodes. No statistically significant difference between the absolute thetapower and the AQ of OEs for SVA was observed (Figure 2A, top panel). As shown in Figure 2B, middle panel, a significant correlation was observed between the absolute spectral power of the gamma band and the AQ of OEs for SAA at the Fz (*r* = 0.54; *p* = 0.047) and F4 (*r* = 0.59; *p* = 0.032) electrodes. As shown in Figure 2A, bottom panel, a negative correlation between the MI of TGC and the AQs of OEs for SVA was observed in the frontal (Fz: *r* = −0.59, *p* = 0.002; FP1: *r* = −0.55, *p* = 0.004; F8: *r* = −0.41, *p* = 0.036), temporal (T6: *r* = −0.51, *p* = 0.008), and central (Cz: *r* = −0.59, *p* = 0.001) regions. The results are summarized in Supplementary Table 2.

**Figure 3:**
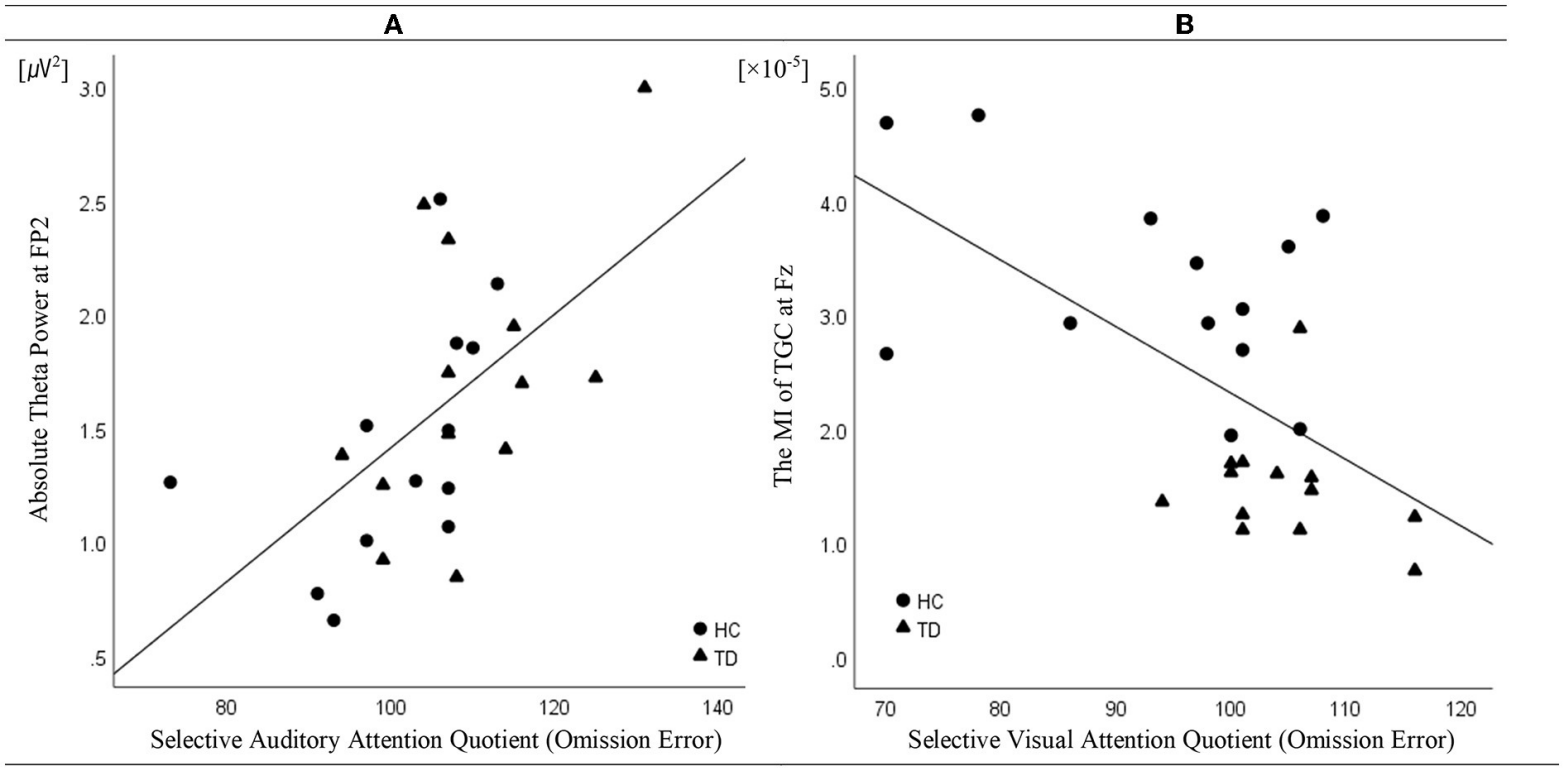
Correlation (linear fit) **(A)** between absolute theta power at FP2 in the resting state and the attention quotients (AQ) of omission errors (OEs) for selective auditory attention in patients with tic disorder and healthy children (*n* = 26, *R*^2^ linear = 0.42, *p* = 0.043) and **(B)** between the modulation index of theta–gamma coupling at Fz during the resting state and the AQ of the OEs for selective visual attention in patients with tic disorder and healthy children (*n* = 26, *R*^2^ linear = 0.43, *p* = 0.001).

Linear regression analysis conducted to determine whether the absolute theta gamma power during the resting state was associated with the OEs for SAA independently of age and sex revealed a significant regression equation [*F*_(3, 25)_ = 5.23; *p* = 0.007] with an *R*^2^ of 0.42. In the linear regression models, the AQ of OEs for SAA was independently and positively associated with the absolute theta power during the resting state at the FP2 (*p* = 0.043) electrode, but not with age (*p* = 0.087) and sex (*p* = 0.557). The absolute theta power during the resting state was the significant predictor of SAA in the model (β = 0.41; *p* = 0.043) (Table 2; Figure 3A).

**Table 2:** Linear regression models of the SAAQ OE as predicted by the absolute theta power at FP2.

	**β**	**B (SE)**	***T***	***p***
Model				0.007
(Constant)		107.56 (11.9)	9.05	0.000
Age	−0.34	−1.10 (0.6)	−1.79	0.087
Sex	−0.10	−2.45 (4.1)	−0.60	0.557
Theta (FP2)	0.41	7.92(3.7)	2.15*	0.043

Linear regression analyses were performed with SAAQ OE as the dependent variable and age, sex, and absolute theta power as independent variables. SAAQ, selective auditory attention quotient; OE, omission error; β, standardized regression coefficient; B, unstandardized coefficientl; SE, standard error. *p < 0.05.

Linear regression analysis conducted to determine whether the MI of TGC was associated with the AQ of OEs for SVA independently of age and sex revealed a significant regression equation [*F*_(3, 25)_ = 5.58; *p* = 0.005] with an *R*^2^ of 0.43. In the linear regression models, the AQ of OE for SVA was independently and negatively associated with the MI of TGC at the Fz (*p* = 0.001) electrode, but not with age (*p* = 0.740) and sex (*p* = 0.086). TGC was the significant predictor of SVA in the model (β = −0.69; *p* = 0.001) (Table 3; Figure 3B). Figure 3 illustrates the correlations of the AQs of OEs for selective attention with absolute theta power and TGC.

**Table 3:** Linear regression analyses were performed with SVAQ OE as the dependent variable and age, sex, and TGC as independent variables.

	**β**	**B (SE)**	***T***	***p***
Model				0.005
(Constant)		103.62 (8.4)	12.37	0.001
Age	0.06	0.18 (0.5)	0.34	0.740
Sex	0.31	7.85 (4.4)	1.80	0.086
TGC (Fz)	−0.69	−698,930.39 (173,384.0)	−4.03*	0.001

SVAQ: selective visual attention quotient, OE: omission error, β: standardized regression coefficient, B: unstandardized coefficient, SE: standard error, TGC: theta–gamma coupling. *p < 0.05.

## Discussion

TGC has been suggested to play a functional role in multi-scale neuronal communication as a neural correlate that coordinates between large-scale functional brain networks and local cortical processing regions (Jensen and Colgin, 2007). Additionally, changes in TGC at the cortical level have been suggested to result in altered resting-state and task-induced activation and deactivation. As TGC is thought to reflect the pattern of signal interaction between active neuron clusters in the brain network (Ahn, 2022; Ahn et al., 2022), the hypoconnectivity of the brain regions constituting the resting-state network of patients with TD may result in abnormal resting TGC. We believe this is the first study to analyze resting-state TGC for investigating large-scale functional interactions in the brains of patients with TD and evaluate the correlation of resting-state TGC with attention. We performed resting-state EEG power spectral and TGC analyses to determine the participants' baseline neurophysiological status. Consistent with the results of a previous study (Ahn, 2022; Ahn et al., 2022), our findings differed between the resting-state spectral and TGC analyses. In the power spectral analysis, the absolute theta and gamma power in some regions was higher in the TD group than in the control group and there were significant between-group differences in absolute gamma power. Gamma bands might be related to impaired inhibitory function and impulsivity and reflect physiological compensation in patients with TD. Conversely, in the TGC analysis, topographical differences were noted in the whole area. This difference in topographical pattern between the two analyses might be because the TGC analysis assesses the amount of information gathered from interacting functional systems across multiple spatiotemporal scales, whereas the spectral analysis evaluates the degree of excitation of the functional neuronal system (Sauseng and Klimesch, 2008; Lee and Jeong, 2013). Patients with TD harbor various atypical networks (Openneer et al., 2020). For example, pediatric patients with TD have hypoconnectivity, immature connectivity, and altered connectivity in their cortical circuits, including the frontoparietal, posterior, and default mode networks, relative to otherwise healthy participants (Wen et al., 2017; Openneer et al., 2020). Resting-state activity is observed in various networks, and the thoughts that spontaneously occur when a person tries to remain awake as they rest with their eyes closed may result from concurrent and extensive co-activation of the default mode network, frontal parietal network, visual network, auditory network, and sensorimotor network (Ong et al., 2015). In our study, the MI of TGC in the resting state was significantly lower in patients with TD than in healthy children, with significant differences (*p* < 0.001) observed in the frontal (Fz, F3, F7, and F6), temporal (T3), central (Cz), parietal (Pz and P4), and occipital (O2) regions. These findings are similar to those from positron emission tomography studies demonstrating that patients with TD have reduced activity in various cortical and subcortical networks (Braun et al., 1993). Our findings add to recent evidence that TD is linked to dysfunctional interactions among multiple brain regions (Stevens et al., 1996; Wen et al., 2017; Openneer et al., 2020) and that resting-state TGC can provide information about the balance between large-scale functional brain networks (Kim et al., 2016; Ahn, 2022; Ahn et al., 2022).

The absolute theta power, absolute gamma power, and TGC during resting-state EEG were assessed to elucidate their relationships with the cognitive function of patients with TD. We found that the CAT selective attention subtest performance, but not the AQ of CEs for SVA, was better in patients with TD than in healthy children. The patients with TD had significantly fewer OEs (indicating that they were less inattentive) than the healthy children. Although the difference was not statistically significant, the patients with TD had more CEs on the SVA test (indicating more impulsivity) than the healthy children. Furthermore, the patients with TD showed fewer OEs and CEs on the SAA test than the healthy children. Patients with TD who exhibit many CEs during the SVA test likely have difficulty ignoring distracting visual stimuli during the resting state with eyes closed. This is in line with previous reports of sensory hypersensitivity in patients with TD (Cohen and Leckman, 1992; Kane, 1994; Harris et al., 1995; Isaacs and Riordan, 2020). When examining the association between resting-state power spectra and CAT selective attention subtest performance, we observed that the absolute theta powers were strongly correlated with the SAA test performance in terms of OEs. In performing multiple logistic regression analysis with adjustment for age and sex, the effect of theta oscillation on the AQ of OEs for SAA remained significant at FP2. Because theta activity may be linked to the inhibition of anticipated and avoidable distractors, an increase in absolute theta power in the resting state may result from increased conscious attention to auditory stimuli due to impaired inhibition function (Schroeder and Lakatos, 2009; Schroeder et al., 2018). Conversely, the hypothesis that a weaker resting-state TGC would be related to poorer performance during the selective attention portion of the CAT subtests was disproved. The AQ of CEs for SVA demonstrated a negative correlation with TGC, indicating that the better the performance on the SVA in terms of OEs, the weaker the synchronization during the resting state with eyes closed. Because sensory attenuation of visual stimuli in the resting state with eyes closed has been reported (Geller et al., 2014; Villena-González et al., 2018), this unexpected result could explain how patients with TD may show abnormally enhanced arousal responses to visual stimuli, particularly in the form of hypersensitivity to visual stimuli (Pareés et al., 2014; Kleimaker et al., 2020). Our results suggest that patients with TD may have abnormal visual sensory attenuation while their eyes are closed. Resting-state brain activity regulates top-down selective attention and arousal, which adequately prepares brain networks for performing WM tasks (Sala-Llonch et al., 2012). Because the top-down modulation of sensory processing relies on long-range inputs and interactions with the frontoparietal network (Giesbrecht et al., 2003; Han and Humphreys, 2007), the observed hypersensitivity to visual stimuli in patients with TD might have been caused by reduced functional long-range connectivity. Our data indicate that TGC may reflect a mechanism of information transfer among functionally connected networks with high inter-regional correlations. It is conceivable that the abnormal reduction of TGC may be linked to dysfunctional interactions related to the attention system at the multi-scale large-network level, which indicates that the arousal responses to sensory stimuli may be related to top-down attention control mechanisms (Kim et al., 2016; Helfrich et al., 2019). These findings support the assumption that patients with TD may have difficulties toning down or gating sensory information.

In here, we confirmed (1) the important role that TGC plays in visual processing in patients with TD and (2) that the mechanisms underlying selective attention differ between the visual and auditory modalities. Finally, we confirmed that the functional interactions among the brain networks of patients with TD differ from those of healthy children even in the resting state (tic-free interval or inter-tic interval). This study has several limitations that must be acknowledged. First, the sample size was small; therefore, the generalizability of our findings may be limited. Second, there was a difference in the distribution of sexes between the TD and control groups. Therefore, replication studies with large, independent, and well-matched samples and sophisticated experimental designs are necessary. Third, approximately half of the patients with TD in this study used antipsychotic medications, which may have influenced the neural activity reflected as the absolute theta power and TGC. To minimize this effect, the patients were instructed to stop using their medication for 2 days before participating in the experiment. Fourth, some children with TD had comorbid conditions; however, our results cannot be explained by comorbidity. Although the patients were instructed to maintain a resting state and not suppress their tics, we cannot exclude the involvement of other cognitive processes for suppressing the patients' tics. Advantages of the EEG data over other neuroimaging data include a better temporal resolution and direct measurement of neural oscillations; in contrast, well-known limitations of EEG are its low spatial precision and inability to measure subcortical activity. The role of subcortical sources remains unclear because the identification of deep brain activity using scalp recordings is controversial. TGC is a possible mechanism underlying the transfer of information from multi-scale brain networks to local cortical processing regions. However, few studies have focused on relationships between cognitive function and the functional role of TGC in the resting state with eyes closed. This study demonstrated that power spectra and TGC analyses may evaluate different aspects of cognitive function and that the degree of interaction between active neuron clusters of brain networks can be assessed. It also demonstrated that SVA test performance, as measured using OEs, is negatively correlated with theta phase synchronization in the resting state when the eyes are closed.

TD may be linked to abnormal enhancement of arousal responses to visual stimuli, particularly hypersensitivity to visual stimuli. Our results demonstrate that patients with TD may exhibit abnormal visual sensory attenuation when their eyes are closed. Furthermore, this study elucidates that cognitive dysfunction is reflected in the abnormal neural activity and hypoconnectivity that form the core of TD pathophysiology. Sensory hypersensitivity to external visual stimuli is likely a clinical manifestation of sensory gating dysfunction. This potential pathophysiologic basis of sensory hypersensitivity aligns with the broader disease model of cortico-striato-thalamo-cortical (CSTC) circuit (Isaacs and Riordan, 2020). Generally, both structural and functional abnormalities within and between circuits can be characterized by a disrupted cognitive control, impulsive behaviors or attention to internal and external stimuli. Clinical features of psychiatric illnesses are rooted in altered activity within relevant brain networks. With the emergence of anatomically selective brain stimulation technologies as therapeutic tools, it is becoming possible to target specific brain circuits in a precise manner, and to modulate their activity in various ways. Techniques for therapeutic brain stimulation including transcranial direct current stimulation, repetitive transcranial magnetic stimulation and deep brain stimulation are entering clinical use for treatment-resistant psychiatric illnesses (Orth and Münchau, 2013; Peters et al., 2016). A study on sensory hypersensitivity could enhance understanding of TD pathophysiology mechanisms and possible treatment implications. Future research is needed to clarify the role of sensory hypersensitivity to external sensory input on underlying motor abnormalities.

## Data availability statement

The original contributions presented in the study are included in the article/Supplementary material, further inquiries can be directed to the corresponding author/s.

## Ethics statement

The study was reviewed and approved by the Board of the Catholic Kwandong University International St. Mary's Hospital in accordance with the Helsinki Declaration. Each subject or parent provided written, informed consent to participate in the study after being informed about the purpose and procedures involved (IRB No. IS21OISI0016). Written informed consent to participate in this study was provided by the participants' legal guardian/next of kin.

## Author contributions

JP: conceptualization, resources, project administration, and funding acquisition. JA: methodology, formal analysis, investigation, data curation, writing—original draft preparation, and visualization. JA and JH: software. JO and KJ: validation. JA, JO, and KJ: writing—review and editing. JP and J-JK: supervision. All authors have read and agreed to the published version of the manuscript.

## Funding

This work was supported by the National Research Foundation of Korea (NRF) grant funded by the Korean government (MSIT) (No. NRF2019R1A2C4069598). This work was also supported by the Korea Medical Device Development Fund grant funded by the Korea government (the Ministry of Science and ICT, the Ministry of Trade, Industry and Energy, the Ministry of Health & Welfare, the Ministry of Food and Drug Safety) (Project Numbers: 1711138277 and KMDF_PR_20200901_0143). The funders had no role in the study design, data collection, and analysis, decision to publish, or preparation of the manuscript.

## Conflict of interest

The authors declare that the research was conducted in the absence of any commercial or financial relationships that could be construed as a potential conflict of interest.

## Publisher's note

All claims expressed in this article are solely those of the authors and do not necessarily represent those of their affiliated organizations, or those of the publisher, the editors and the reviewers. Any product that may be evaluated in this article, or claim that may be made by its manufacturer, is not guaranteed or endorsed by the publisher.

## Abbreviations

TD: tic disorder
TGC: theta–gamma coupling
WM: working memory
EEG: electroencephalography
SVA: selective visual attention
SAA: selective auditory attention
MI: modulation index
CAT: comprehensive attention test
OE: omission error
CE: commission error
AQ: attention quotient
FDR: false discovery rate
CSTC: cortico-striato-thalamo-cortical.
