# Supplementary material for: Association of resting-state theta–gamma coupling with selective visual attention in children with tic disorders
Source: Front Hum Neurosci. 2022 Sep 29;16:1017703. doi: 10.3389/fnhum.2022.1017703 (PMC9558697; doi:10.3389/fnhum.2022.1017703)
Supplement: Supplementary file 1 [file Data_Sheet_1.docx]

Supplementary Material

# Supplementary Tables

**Supplementary Table 1.** The comparisons of absolute power and Theta-Gamma Coupling between HC and patients with TD

| Lead | | TD | | HC | | t | p_FDR_ |
| --- | --- | --- | --- | --- | --- | --- | --- |
| (㎶) | | Mean | SD | Mean | SD |  |  |
| Absolute theta power | FP2 | 1.71 | 0.61 | 1.43 | 0.54 | 1.21 | 0.322 |
|  | Fz | 1.66 | 0.63 | 1.43 | 0.55 | 1.00 | 0.385 |
|  | FP1 | 1.78 | 0.57 | 1.43 | 0.56 | 1.56 | 0.244 |
|  | F3 | 1.47 | 0.41 | 1.25 | 0.48 | 1.22 | 0.322 |
|  | F7 | 1.52 | 0.56 | 1.18 | 0.35 | 1.87 | 0.223 |
|  | C3 | 1.15 | 0.39 | 0.88 | 0.32 | 1.93 | 0.223 |
|  | T3 | 1.44 | 0.66 | 0.96 | 0.38 | 2.27 | 0.223 |
|  | P3 | 1.18 | 0.52 | 0.89 | 0.42 | 1.59 | 0.244 |
|  | T5 | 1.13 | 0.48 | 0.85 | 0.40 | 1.59 | 0.244 |
|  | Pz | 1.02 | 0.52 | 0.92 | 0.47 | 0.51 | 0.641 |
|  | O1 | 1.18 | 0.59 | 1.11 | 0.46 | 0.32 | 0.741 |
|  | O2 | 1.29 | 0.86 | 0.94 | 0.50 | 1.27 | 0.322 |
|  | P4 | 1.00 | 0.54 | 0.83 | 0.41 | 0.94 | 0.395 |
|  | T6 | 1.38 | 0.76 | 0.92 | 0.53 | 1.81 | 0.223 |
|  | C4 | 1.00 | 0.37 | 0.83 | 0.34 | 1.21 | 0.322 |
|  | T4 | 1.30 | 0.47 | 0.89 | 0.39 | 2.40 | 0.223 |
|  | F8 | 1.40 | 0.51 | 1.09 | 0.33 | 1.88 | 0.223 |
|  | F4 | 1.39 | 0.48 | 1.08 | 0.33 | 1.95 | 0.223 |
|  | Cz | 0.95 | 0.49 | 1.14 | 0.50 | -1.00 | 0.385 |
| Absolute gamma power | FP2 | 0.22 | 0.05 | 0.20 | 0.05 | 1.63 | 0.118 |
|  | Fz | 0.21 | 0.04 | 0.17 | 0.05 | 2.46* | 0.022 |
|  | FP1 | 0.25 | 0.04 | 0.18 | 0.05 | 3.71* | 0.001 |
|  | F3 | 0.21 | 0.05 | 0.15 | 0.03 | 3.68* | 0.001 |
|  | F7 | 0.23 | 0.06 | 0.15 | 0.02 | 4.34* | 0.001 |
|  | C3 | 0.17 | 0.04 | 0.10 | 0.02 | 5.46^†^ | <0.001 |
|  | T3 | 0.25 | 0.08 | 0.15 | 0.05 | 3.66* | 0.001 |
|  | P3 | 0.17 | 0.06 | 0.10 | 0.03 | 3.64* | 0.002 |
|  | T5 | 0.15 | 0.03 | 0.10 | 0.03 | 4.48* | 0.001 |
|  | Pz | 0.16 | 0.06 | 0.09 | 0.03 | 3.54* | 0.002 |
|  | O1 | 0.23 | 0.10 | 0.13 | 0.04 | 3.24* | 0.004 |
|  | O2 | 0.24 | 0.16 | 0.12 | 0.06 | 2.55* | 0.019 |
|  | P4 | 0.13 | 0.04 | 0.08 | 0.02 | 3.88* | 0.001 |
|  | T6 | 0.20 | 0.09 | 0.10 | 0.05 | 3.70* | 0.001 |
|  | C4 | 0.13 | 0.03 | 0.10 | 0.02 | 4.44* | 0.001 |
|  | T4 | 0.26 | 0.10 | 0.12 | 0.04 | 4.49* | 0.001 |
|  | F8 | 0.21 | 0.05 | 0.15 | 0.04 | 3.37* | 0.003 |
|  | F4 | 0.18 | 0.03 | 0.13 | 0.02 | 4.31* | 0.001 |
|  | Cz | 0.12 | 0.03 | 0.11 | 0.03 | 0.91 | 0.357 |
| Theta-Gamma Coupling | FP2 | 1.46 | 0.68 | 3.41 | 0.51 | 8.26* | 0.010 |
|  | Fz | 1.49 | 0.50 | 3.26 | 0.88 | 6.27^†^ | <0.001 |
|  | FP1 | 1.60 | 0.54 | 3.38 | 0.33 | 10.08* | 0.030 |
|  | F3 | 1.40 | 0.50 | 3.63 | 1.69 | 4.55^†^ | <0.001 |
|  | F7 | 1.29 | 0.32 | 3.08 | 0.55 | 10.14* | 0.030 |
|  | C3 | 1.43 | 0.36 | 3.29 | 0.64 | 9.10* | 0.018 |
|  | T3 | 1.37 | 0.34 | 3.81 | 2.59 | 3.36* | 0.004 |
|  | P3 | 1.35 | 0.50 | 3.25 | 0.79 | 7.33* | 0.004 |
|  | T5 | 1.40 | 0.60 | 3.54 | 2.60 | 2.91* | 0.009 |
|  | Pz | 1.57 | 0.49 | 3.01 | 0.67 | 6.25^†^ | <0.001 |
|  | O1 | 1.30 | 0.35 | 3.22 | 0.74 | 8.49* | 0.012 |
|  | O2 | 1.63 | 0.64 | 3.40 | 1.13 | 4.95^†^ | <0.001 |
|  | P4 | 1.67 | 0.52 | 3.04 | 0.95 | 4.56^†^ | <0.001 |
|  | T6 | 1.53 | 0.47 | 3.49 | 0.57 | 9.57* | 0.024 |
|  | C4 | 1.40 | 0.41 | 3.27 | 0.88 | 6.96* | 0.003 |
|  | T4 | 1.37 | 0.54 | 3.34 | 0.95 | 6.49* | 0.002 |
|  | F8 | 1.26 | 0.39 | 3.03 | 0.86 | 6.78* | 0.002 |
|  | F4 | 1.64 | 0.61 | 3.73 | 0.86 | 7.18* | 0.004 |
|  | Cz | 1.61 | 0.66 | 3.39 | 0.86 | 5.91^†^ | <0.001 |

HC: Healthy children, TD: Tic disorder, SD: Standard deviation, FDR: False Discovery Rate, TGC: Theta-Gamma Coupling. *p<0.05, †p<0.001.

**Supplementary Table 2.** Pearson’s correlation coefficients between the and clinical characteristics across HC and patients with TD

|  | Absolute theta power | | | | absolute gamma power | | | | Theta-Gamma Coupling | | | |
| --- | --- | --- | --- | --- | --- | --- | --- | --- | --- | --- | --- | --- |
| Lead | VSAQ OE | | ASAQ OE | | VSAQ OE | | ASAQ OE | | VSAQ OE | | ASAQ OE | |
|  | r | *p*_FDR_ | R | *p*_FDR_ | r | *p*_FDR_ | r | *p*_FDR_ | r | *p*_FDR_ | r | *p*_FDR_ |
| FP2 | 0.15 | 0.466 | 0.57* | 0.013 | 0.37 | 0.405 | 0.44 | 0.099 | -0.25 | 0.213 | -0.40 | 0.199 |
| Fz | 0.11 | 0.584 | 0.60* | 0.012 | 0.38 | 0.405 | 0.54* | 0.047 | -0.59* | 0.002 | -0.19 | 0.385 |
| FP1 | 0.11 | 0.578 | 0.56* | 0.013 | 0.25 | 0.469 | 0.50 | 0.055 | -0.55* | 0.004 | -0.39 | 0.199 |
| F3 | 0.06 | 0.756 | 0.49* | 0.034 | 0.36 | 0.405 | 0.30 | 0.318 | -0.36 | 0.070 | -0.29 | 0.240 |
| F7 | 0.14 | 0.505 | 0.53* | 0.021 | 0.34 | 0.405 | 0.30 | 0.318 | -0.33 | 0.097 | -0.27 | 0.240 |
| C3 | 0.00 | 0.998 | 0.44 | 0.055 | 0.22 | 0.469 | 0.27 | 0.318 | -0.23 | 0.269 | -0.39 | 0.199 |
| T3 | 0.05 | 0.826 | 0.37 | 0.115 | 0.20 | 0.469 | -0.02 | 0.936 | -0.25 | 0.228 | -0.32 | 0.238 |
| P3 | -0.07 | 0.722 | 0.32 | 0.138 | 0.23 | 0.469 | 0.16 | 0.543 | -0.26 | 0.200 | -0.45 | 0.190 |
| T5 | -0.09 | 0.678 | 0.34 | 0.124 | 0.17 | 0.469 | 0.16 | 0.543 | -0.17 | 0.402 | -0.34 | 0.231 |
| Pz | 0.02 | 0.918 | 0.28 | 0.192 | 0.29 | 0.440 | 0.16 | 0.543 | -0.34 | 0.093 | -0.21 | 0.373 |
| O1 | -0.12 | 0.581 | 0.20 | 0.356 | 0.18 | 0.469 | 0.15 | 0.543 | -0.37 | 0.064 | -0.35 | 0.231 |
| O2 | -0.07 | 0.708 | 0.45 | 0.054 | 0.15 | 0.502 | 0.45 | 0.095 | -0.36 | 0.068 | -0.09 | 0.660 |
| P4 | -0.05 | 0.820 | 0.33 | 0.133 | 0.16 | 0.490 | 0.25 | 0.340 | -0.10 | 0.620 | -0.51 | 0.147 |
| T6 | 0.00 | 0.998 | 0.17 | 0.408 | 0.20 | 0.469 | 0.05 | 0.842 | -0.51* | 0.008 | -0.31 | 0.238 |
| C4 | -0.06 | 0.769 | 0.40 | 0.081 | 0.18 | 0.469 | 0.28 | 0.318 | -0.30 | 0.135 | -0.27 | 0.240 |
| T4 | 0.01 | 0.979 | 0.34 | 0.124 | 0.27 | 0.440 | 0.11 | 0.650 | -0.25 | 0.213 | -0.33 | 0.236 |
| F8 | 0.15 | 0.453 | 0.45 | 0.054 | 0.30 | 0.440 | 0.29 | 0.318 | -0.41* | 0.036 | -0.30 | 0.239 |
| F4 | 0.11 | 0.602 | 0.61* | 0.012 | 0.28 | 0.440 | 0.59* | 0.032 | -0.38 | 0.056 | -0.19 | 0.385 |
| Cz | -0.19 | 0.365 | 0.35 | 0.124 | 0.04 | 0.848 | 0.41 | 0.121 | -0.59* | 0.001 | -0.27 | 0.240 |

Values are Pearson’s correlation coefficients. *p<0.05. HC: Healthy Children, TD: Tic Disorders, SVAQ: Selective Visual Attention Quotient, OE: Omission Error, SAAQ: Selective Auditory Attention Quotient, FDR: False Discovery Rate.
